# Supplementary material for: Clinical Evidence for the Importance of the Wild-Type PRPF31 Allele in the Phenotypic Expression of RP11
Source: Genes (Basel). 2021 Jun 14;12(6):915. doi: 10.3390/genes12060915 (PMC8232116; doi:10.3390/genes12060915)
Supplement: Supplementary file 1 [file genes-12-00915-s001.zip › genes-1233424-supplementary.pdf]

Supplementary materials

Clinical evidence of implication of wild-type *PRPF31* allele in phenotypic expression of RP11

Danial Roshandel, Jennifer A. Thompson, Rachael C. Heath Jeffery, Dan Zhang, Tina M. Lamey, Terri L. McLaren, John N. De Roach, Samuel McLenachan, David A. Mackey and Fred K. Chen

Table S1. Detection and classification of variants.

Table S2. Baseline functional measures.

Table S3. Baseline structural measures.

Table S4. Longitudinal changes in MAIA 10-2 and 37R mean sensitivity.

Table S5. Longitudinal changes in residual EZ span and NIAF HAR area.

Figure S1. MAIA patterns.

Figure S2. Clinical findings in non-penetrant carrier.

Figure S3. Clinical findings in patient 1313.

Figure S4. Visual field findings in members of family 0255.

Table S1. Detection and classification of variants detected in RP11 families.

AIRDR Ped ID	WARD study ID	Testing methodology	*PRPF31* Variant	ACMG Pathogenicity classification	Refs or Novel
0255	1313	Targeted Sanger Seq	c.267del<br>p.(Glu89Aspfs*11)<br>(nonsense-mediated mRNA decay predicted)	Pathogenic	[1]
	1477	Targeted Sanger Seq			
	1150	RD NGS Panel v1 (131 genes)			
	1651	Targeted Sanger Seq			
	1506	Targeted Sanger Seq			
	1332	Targeted Sanger Seq			
3200	1816	Targeted Sanger Seq	c.772_773delins16<br>(ins CAACATGCAACATCAT)<br>p.(Thr258Glnfs*68)<br>(nonsense-mediated mRNA decay predicted)	Pathogenic	[2]
	1757	Targeted Sanger Seq			
	1681	MVL Vision Panel v2 (577 genes)<br>+ CNV analysis			
0244	1473	MVL Vision Panel v1 (537 genes)	c.-9+1G>T<br>p.?<br>Functional evidence of truncated protein	Pathogenic	[3,4]
2097	1708	RD NGS Panel v4 (183 genes)	c.527+1G>T<br>p.(Gly176fs*186)<br>Functional evidence of truncated protein	Pathogenic	[5-8]
0213	1705	Array CGH analysis	Exon 2-3 del<br>c.(?_1)_(238+1_239-1)del<br>(no-go mRNA decay expected)	Likely pathogenic	[9,10]
0725	1164	MVL Vision Panel v2 (577 genes)<br>+ CNV analysis	Exon 2-8 del<br>c.(-9+1_-8-1)_(855+1_856-1)del<br>p.?<br>(no-go mRNA decay expected)	VUS	Novel
0155	1175	Array CGH analysis	Exon 9-14del<br>c.(855+1_?)del<br>p.?<br>(non-stop mRNA decay expected)	VUS	Novel

References

1. Sullivan, L.S.; Bowne, S.J.; Reeves, M.J.; Blain, D.; Goetz, K.; Ndifor, V.; Vitez, S.; Wang, X.; Tumminia, S.J.; Daiger, S.P. Prevalence of mutations in eyeGENE probands with a diagnosis of autosomal dominant retinitis pigmentosa. *Invest Ophthalmol Vis Sci*, 2013. **54**, 6255-61, doi: 10.1167/iovs.13-12605.

2. Zhao, L.; Wang, F.; Wang, H.; Li, Y.; Alexander, S.; Wang, K.; Willoughby, C.E.; Zaneveld, J.E.; Jiang, L.; Soens, Z.T.; et al. Next-generation sequencing-based molecular diagnosis of 82 retinitis pigmentosa probands from Northern Ireland. *Hum Genet*, 2015. **134**, 217-30, doi: 10.1007/s00439-014-1512-7.

3. Audo, I.; Bujakowska, K.; Mohand-Said, S.; Lancelot, M.E.; Moskova-Doumanova, V.; Waseem, N.H.; Antonio, A.; Sahel, J.A.; Bhattacharya, S.S.; Zeitz, C. Prevalence and novelty of PRPF31 mutations in French autosomal dominant rod-cone dystrophy patients and a review of published reports. *BMC Med Genet*, 2010. **11**, 145, doi: 10.1186/1471-2350-11-145.

4. Liu, J.Y.; Dai, X.; Sheng, J.; Cui, X.; Wang, X.; Jiang, X.; Tu, X.; Tang, Z.; Bai, Y.; Liu, M.; et al. Identification and functional characterization of a novel splicing mutation in RP gene PRPF31. *Biochem Biophys Res Commun*, 2008. **367**, 420-6, doi: 10.1016/j.bbrc.2007.12.156.

5. Abdulridha-Aboud, W.; Kjellstrom, U.; Andreasson, S.; Ponjavic, V. Characterization of macular structure and function in two Swedish families with genetically identified autosomal dominant retinitis pigmentosa. *Mol Vis*, 2016. **22**, 362-73,

6. Chakarova, C.F.; Cherninkova, S.; Tournev, I.; Waseem, N.; Kaneva, R.; Jordanova, A.; Veraitch, B.K.; Gill, B.; Colclough, T.; Nakova, A.; et al. Molecular genetics of retinitis pigmentosa in two Romani (Gypsy) families. *Mol Vis*, 2006. **12**, 909-14,

7. Kiser, K.; Webb-Jones, K.D.; Bowne, S.J.; Sullivan, L.S.; Daiger, S.P.; Birch, D.G. Time Course of Disease Progression of PRPF31-mediated Retinitis Pigmentosa. *Am J Ophthalmol*, 2019. **200**, 76-84, doi: 10.1016/j.ajo.2018.12.009.

8. Martin-Merida, I.; Sanchez-Alcudia, R.; Fernandez-San Jose, P.; Blanco-Kelly, F.; Perez-Carro, R.; Rodriguez-Jacy da Silva, L.; Almoguera, B.; Garcia-Sandoval, B.; Lopez-Molina, M.I.; Avila-Fernandez, A.; et al. Analysis of the PRPF31 Gene in Spanish Autosomal Dominant Retinitis Pigmentosa Patients: A Novel Genomic Rearrangement. *Invest Ophthalmol Vis Sci*, 2017. **58**, 1045-1053, doi: 10.1167/iovs.16-20515.

9. Jespersgaard, C.; Fang, M.; Bertelsen, M.; Dang, X.; Jensen, H.; Chen, Y.; Bech, N.; Dai, L.; Rosenberg, T.; Zhang, J.; et al. Molecular genetic analysis using targeted NGS analysis of 677 individuals with retinal dystrophy. *Sci Rep*, 2019. **9**, 1219, doi: 10.1038/s41598-018-38007-2.

10. Xiao, T.; Xie, Y.; Zhang, X.; Xu, K.; Zhang, X.; Jin, Z.B.; Li, Y. Variant Profiling of a Large Cohort of 138 Chinese Families With Autosomal Dominant Retinitis Pigmentosa. *Front Cell Dev Biol*, 2020. **8**, 629994, doi: 10.3389/fcell.2020.629994.

Table S2. Baseline functional measures in individuals carrying different mutations.

		HVF 24-2			MAIA 10-2					MAIA 37R				
Mutation	ID	Age (y)	RE MD (dB)	LE MD (dB)	Age (y)	RE MS (dB)	LE MS (dB)	RE SL (n)	LE SL (n)	Age (y)	RE MS (dB)	LE MS (dB)	RE SL (n)	LE SL (n)
c.267del	1332	-	-	-	-	-	-	-	-	-	-	-	-	-
	1506	-	-	-	34	19.1	17.8	68	68	34	29.1	28.2	37	37
	1150	44	-26.9	-26.7	-	-	-	-	-	49	4.7	9.4	32	35
	1477	61	-30.3	-30.5	-	-	-	-	-	-	-	-	-	-
	1313	-	-	-	-	-	-	-	-	-	-	-	-	-
c.772_773delins16	1681	16	-12.0	-15.5	16	19.8	20.0	68	65	16	28.4	25.8	37	37
	1757	18	-3.7	-2.6	19	29.8	29.9	68	68	19	30.9	31.0	37	37
c.-9+1G>T	1473	52	-28.41	-24.12	-	-	-	-	-	-	-	-	-	-
c.527+1G>T	1708	-	-	-	-	-	-	-	-	-	-	-	-	-
Exon 2-3del	1705	-	-	-	-	-	-	-	-	62	8.2	9.8	28	30
Exon 2-8del	1164	61	-28.1	-28.1	61	8.5	7.2	43	43	61	23.3	23.5	37	37
Exon 9-14del	1175	38	-27.6	-28.0	35	1.4	0	22	15	35	15.2	8.5	34	31

HVF = Humphrey visual field; LE = left eye; MD = mean deviation; MS = mean sensitivity; RE = right eye; SL = seeing loci.

Table S3. Baseline structural measures in individuals carrying different mutations.

		Single line scan EZ length (μm)			NIAF HAR area (mm^2)			SWAF HAR area (mm^2)		
Mutation	ID	Age (y)	RE	LE	Age (y)	RE	LE	Age (y)	RE	LE
c.267del	1332	29	2220	2554	-	-	-	-	-	-
	1506	34	4368	3967	34	14.3	12.1	34	16.1	13.1
	1150	44	0	0	51	0	0	51	0	0
	1477	61	1505*	2529*	-	-	-	-	-	-
	1313	81	0	0	-	-	-	-	-	-
c.772_773delins16	1681	16	4219	4428	16	14.2	15.2	16	NM‡	NM‡
	1757	18	NM**	NM**	18	NM**	NM**	18	NM**	NM**
c.-9+1G>T	1473	63	4945†	4112	69	0†	5.5	69	0†	6.0
c.527+1G>T	1708	70	0	0	70	0	0	70	0	0
Exon 2-3del	1705	62	0	753	62	0	0	62	0	0
Exon 2-8del	1164	61	3553	3395	61	9.5	8.3	61	10.8	9.2
Exon 9-14del	1175	35	1014*	0	35	2.1	0	35	2.6	0

*Attenuated and interrupted EZ line **Boundaries fell beyond imaging field †Distorted image due to post-penetrating keratoplasty high irregular astigmatism ‡Borders very blurred

EZ = ellipsoid zone; HAR = hyperautofluorescent ring; LE = left eye; NIAF = near-infrared autofluorescence; NM = not measurable; RE = right eye; SWAF = short-wavelength autofluorescence.

Table S4. Longitudinal changes in MAIA 10-2 and 37R mean sensitivity.

			MAIA 10-2 MS (dB)							MAIA 37R MS (dB)						
Mutation	ID	Eye	Exams (n)	Baseline age (y)	FU (y)	Baseline	Final	Slope (dB/y)	Change (%/y)	Exams (n)	Baseline age (y)	FU (y)	Baseline	Final	Slope (dB/y)	Change (%/y)
c.772_773delins16	1681	RE	3	16	1.0	19.8	22.0	+2.2	+11.1	3	16	1.0	28.4	30.5	+2.1	+7.4
	1757	RE	2	19	0.5	29.8	30.9	+2.2	+7.4	2	19	0.5	30.9	32.6	+3.4	+11.0
Exon 2-8del	1164	RE	7	61	4.3	8.5	10.2	+0.4	+4.7	7	61	4.3	23.3	25.0	+0.4	+1.7
Exon 9-14del	1175	RE	9	35	4.8	1.4	4.0	+0.4*	+28.6	9	35	4.8	15.2	13.3	-0.4*	-2.6

* Significant at $p<0.05$ (linear regression analysis).

FU = follow-up duration; MS = mean sensitivity; RE = right eye.

Table S5. Longitudinal changes in residual EZ span and NIAF HAR area.

			EZ span (µm)							NIAF HAR area (mm^2)						
Mutation	ID	Eye	Exams (n)	Baseline age (y)	FU (y)	Initial	Final	Slope (µm/y)	Change (%/year)	Exams (n)	Baseline age (y)	FU (y)	Initial	Final	Slope (mm^2/y)	Change (%/year)
c.772_773delins16	1681	RE	4	16	1.5	4519	4308	-142	-3.1	4	16	1.5	14.6	13.7	-0.3	-2.1
c.-9+1G>T	1473	LE	13	63	8.9	4112	2555	-174*	-4.2	6	69	2.6	5.5	4.5	-0.4*	-7.2
Exon 2-3del	1705	LE	2	62	1.1	753	632	-112	-14.9	-	-	-	-	-	-	-
Exon 2-8del	1164	RE	9	61	4.5	3553	3252	-72*	-2.0	9	61	4.5	9.5	7.1	-0.6*	-6.3
Exon 9-14del	1175	RE	9	35	4.9	1014	764	-65*	-6.4	9	35	4.9	2.1	1.2	-0.2*	-9.5

* Significant at $p<0.05$ (linear regression analysis).

EZ = ellipsoid zone; FU = follow-up duration; HAR = hyperautofluorescent ring; LE = left eye; NIAF = near-infrared autofluorescence; RE = right eye.

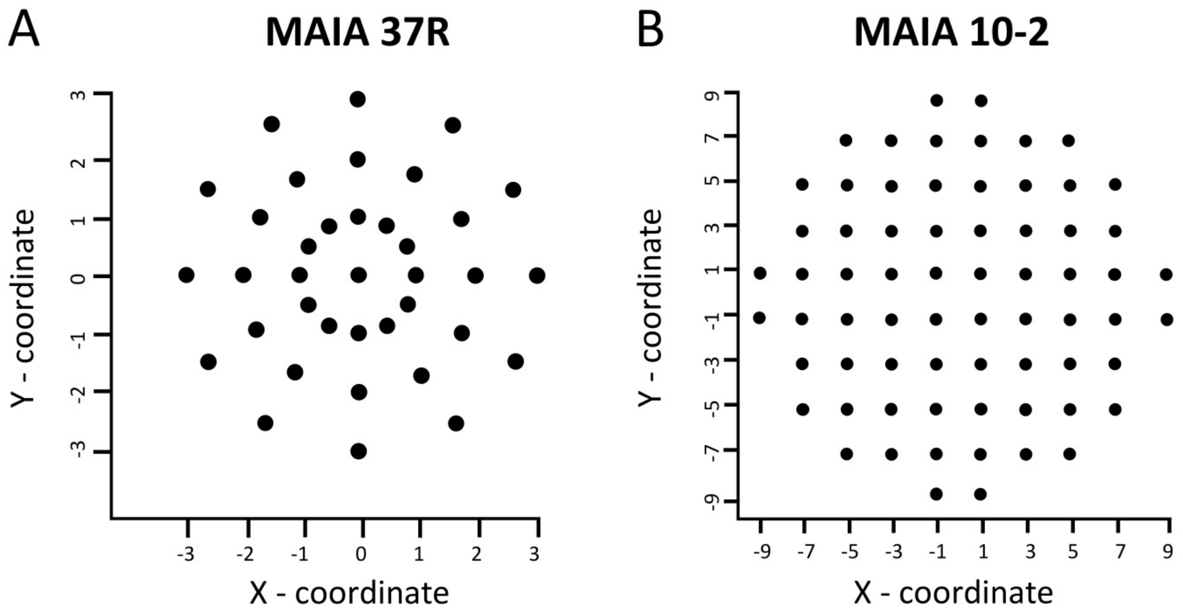

Figure S1. Patterns of microperimetry testing with macular integrity assessment (MAIA). (A) small grid containing 37 radially distributed (37R) test loci spanning 6° of central macula, (B) large grid containing 68 test loci spanning approximately 20° of central macula.

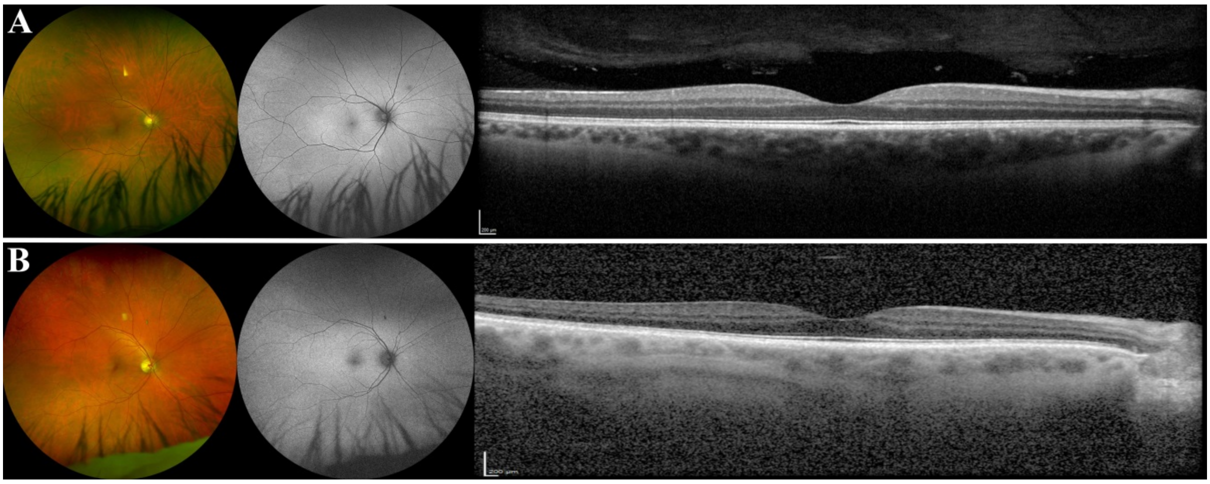

Figure S2. Ultra widefield colour fundus photograph (left) and autofluorescence imaging (middle) and macular spectral-domain optical coherence tomography was unremarkable in non-penetrant carriers with c.267del (A) and c.772_773delins16 (B).

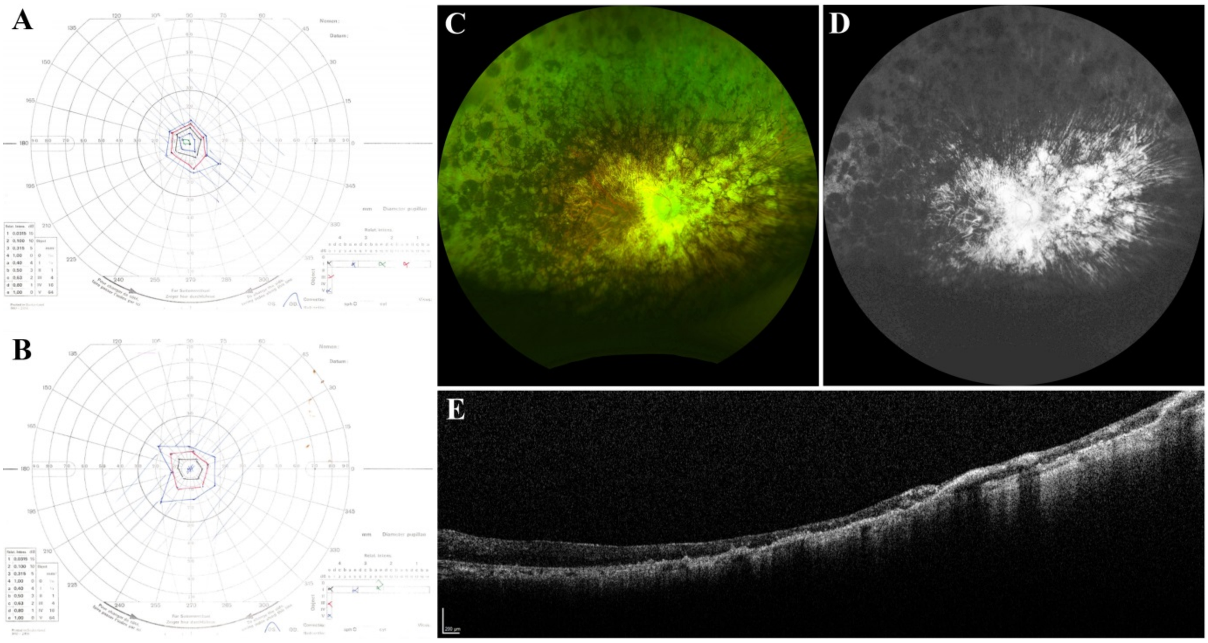

Figure S3. Manual kinetic perimetry of patient 1313 with childhood-onset retinitis pigmentosa shows severe constriction of the peripheral visual field reduced to less than 10° using V4e target (blue lines) in the right (A) and left (B) eyes at age 60 years. Extensive chorioretinal atrophy and retinal pigmentation were detected on ultra widefield fundus photograph (C) and autofluorescence imaging (D), as well as macular spectral-domain optical coherence tomography (E).

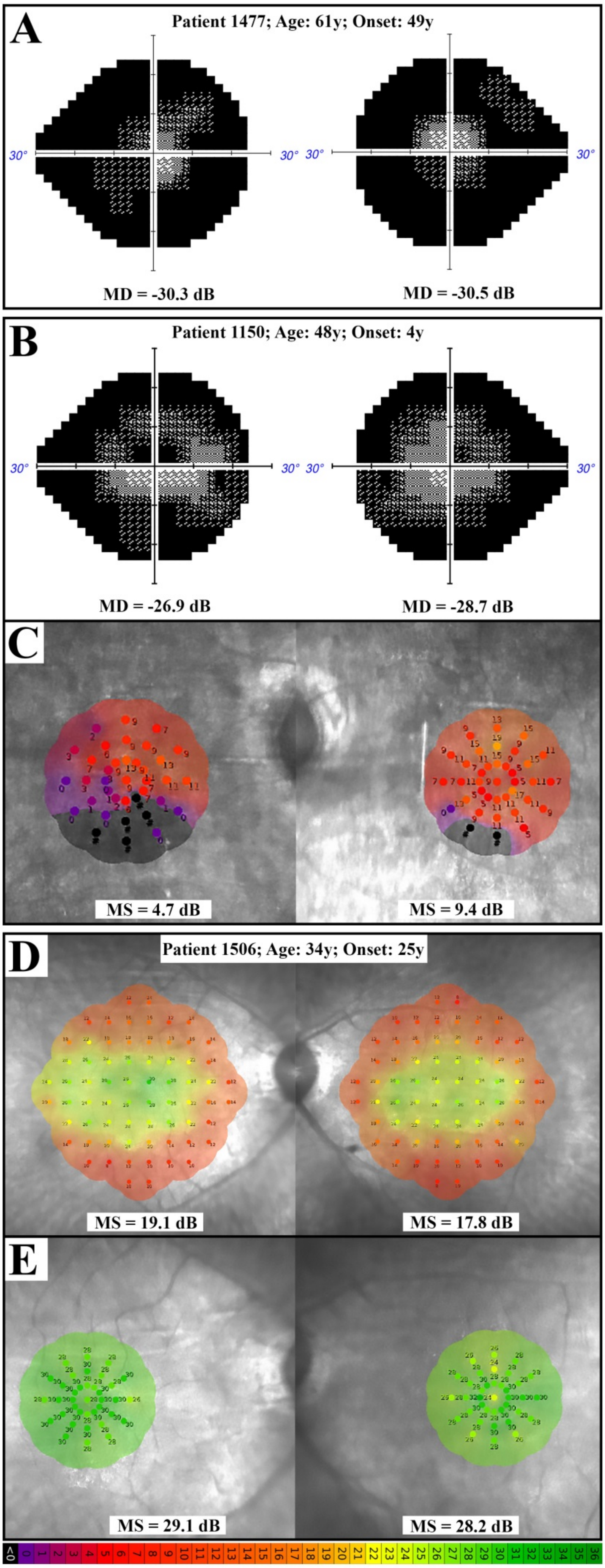

Figure S4. Available perimetry tests in members of family 0255 with c.267del. Humphrey 24-2 visual field test showed severe generalized depression and dense peripheral scotoma with small central island in patients 1477 (A) and 1150 (B), both diagnosed as RP with rapid progression. Foveal (37R) microperimetry showed residual foveal sensitivity in both eyes of patient 1150 (C). The scotoma detected on foveal microperimetry in both eyes is compatible with the scotoma observed in Humphrey 24-2 test (B and C). Patient 1506 with adult-onset disease with slow progression revealed substantial residual macular function (D) and normal foveal sensitivity (E) in both eyes. The colour codes of microperimetry sensitivities in dB unit are shown at the bottom of the panel E.
